# Potential efficacy and mechanisms of icariin for the animal model of osteonecrosis of the femoral head

**DOI:** 10.3389/fphar.2025.1508971

**Published:** 2025-02-19

**Authors:** Jie Xu, Wei Deng, Xun Zhu, Deyu Han, Yu Zheng, Qun Zheng

**Affiliations:** ^1^ Department of Rheumatology Immunology, The Second Affiliated Hospital and Yuying Children’s Hospital of Wenzhou Medical University, Wenzhou, China; ^2^ Department of Internal Medicine, Mianning County People’s Hospital, Wenzhou, China; ^3^ Department of Nephrology, The Second Affiliated Hospital and Yuying Children’s Hospital of Wenzhou Medical University, Wenzhou, China

**Keywords:** icariin, osteonecrosis of the femoral head, efficacy, mechanisms, methodology

## Abstract

**Introduction:**

Icariin (ICA), one of the main active components of *Epimediumis*, is reported to revere osteonecrosis of the femoral head (ONFH). The aim of this study is to further explore the mechanisms and efficacy of ICA in animal models of ONFH and simultaneously analyze methodological issues in the field of researches.

**Methods:**

According to the established search strategy, we searched 14 studies from eight databases from their inception dates to November 2024. The CAMARADES 10-item checklist was utilized to evaluate the methodological quality of the stuies and Rev-Man 5.3 software to analyze differences in outcome indicators.

**Results:**

The quality score for the included articles ranges from 1/10 to 6/10 with an average of 4.5 and the defects in aspects of blinding assessment of outcome, randomization and sample size calculation are the main losing points. Compared with the control group, 10 stuies reported ICA could partially improve bone pathology including reducing the empty of bone lacunae, maturing osteoblasts of ONFH and decreasing chondroid bone matrix and fibrous connective tissue. Eight stuies indicates that ICA could improve bone-related parameters under imageology including F-BMD, Tb.N, Tb.Th, BV/TV and Tb. Sp.

**Discussion:**

The preclinical systematic review provided preliminary evidence that ICA partially reversing ONFH in animal models probably via promoting angiogenesis, anti-apoptosis, and regulating the activities of osteoblasts and osteoclasts. Randomization, blinding and sample size calculation should be focused on in future studies of ONFH. These findings suggest that ICA is a potential candidate for further clinical trials of ONFH.

## 1 Introduction

Osteonecrosis of the femoral head (ONFH) is a destructive disease that often necessitates hip replacement due to pain and dysfunction of the hip joint (Zhao et al., 2020). It most commonly arises from the fracture of the femoral neck, glucocorticoid and alcohol use, but it is also associated with blood dyscrasias and metabolic and coagulation disorders (Fang et al., 2022; George and Lane, 2022). It is estimated that 8.12 million patients with an increasing trend year after year in China suffered from ONFH (Zhao et al., 2015), which places a severe psychological pressure and heavy economic burden on patients (Huang et al., 2020; Liu et al., 2022). Total hip arthroplasty (THA) is recognized as the ultimate therapeutic treatment option for advanced osteoarthritis caused by femoral head collapse (Kuroda et al., 2021). However, complications such as periprosthetic infection and aseptic loosening are non-negligible (Sodhi et al., 2020). Many patients also need to undergo retrofitting surgery due to the limited lifespan of the prosthesis (Kong et al., 2022). Therefore, finding a method that can delay the progression of the disease rather than surgical treatment is usually the first choice in some patients with mild ONFH. Using anticoagulants and vasodilators might be the best first step to treat ONFH step by step. However, the progress of ONFH cannot be halted sometimes (Zhao et al., 2020; Zhang et al., 2020). Although bisphosphonates can significantly improve bone remodeling outcomes in animal models, no significant therapeutic efficacy on ONFH has been observed in clinical studies (Li et al., 2018). Moreover, the risk of atypical fractures caused by long-term inhibition of bone resorption from bisphosphonates should be vigilant. In clinical practice, hip-preserving methods such as core decompression (CD), non-vascularized or vascularized bone grafting, rotational osteotomies, and tantalum rod implantation have achieved a certain degree of effectiveness during the early stages of ONFH (He et al., 2020; Mo et al., 2022). However, the success rate of these preservation treatments listed above was not as effective as expected, and as the collapse of the femoral head, the success rate decreases significantly (Atilla et al., 2020; He et al., 2020; Sadile et al., 2016; Mo et al., 2022). Thus, it is still urgent to find new anti-bone destruction treatment methods to improve the efficiency of mild ONFH treatment.

Icariin (ICA), one of the main active components of *Epimedium*, has immunoregulatory, anti-inflammatory, anti-aging, and anti-bone destruction activities (Li et al., 2020). Because of its potential anti-bone destruction effect, ICA has been selected by many researchers to study its effect on osteoporosis- and osteonecrosis-related diseases (Wang et al., 2022; Gao and Zhang, 2022). Although the pathogenesis of ONFH remains unclear, an imbalance of bone metabolism is regarded as one of the most crucial causes (Li et al., 2018). When ONFH occurs, bone formation fails to keep pace with bone resorption, resulting in low bone mineral density in the femoral head and a progressive collapse (Li et al., 2018). Yang et al. has demonstrated that ICA could effectively enhance the proliferation and osteogenic differentiation of bone marrow stem cells (BMSCs) (Yang et al., 2019). Furthermore, it can also expedite the differentiation and proliferation of osteoblasts (Wang et al., 2018; Teng et al., 2022) and directly inhibit bone resorption by osteoclasts (Kim et al., 2018). Based on the abovementioned theoretical foundation, current investigations (Yu et al., 2019; Huang et al., 2018) were developed and found that ICA could improve ONFH through a variety of pharmacological activity studies. However, the promotion and use of ICA in clinical practice are limited due to a lack of evidence and unknown mechanisms of action. Therefore, this study aims to explore the preclinical evidence and possible mechanisms of ICA in animal models of ONFH.

## 2 Methods

### 2.1 Data sources and search strategies

Comprehensive literature searches about animal studies of ICA for ONFH were conducted in Wanfang, Chinese Science and Technology Journal Database, Chinese Biomedical Database, China National Knowledge Infrastructure, PubMed, Embase, Cochrane Library, and Web of Science database from their inception dates to November 2024. The following search terms were used in PubMed and were modified to suit other databases: “Icariin” AND (“Femur head necrosis” OR “Femoral head necrosis” OR “Osteonecrosis” OR “Osteonecrosis of the Femoral Head”). A complete record of search strings in PubMed is provided as an example in Appendix 1. The reference lists of potential literature sources were manually searched.

### 2.2 Eligibility criteria

Jie Xu and Wei Deng separately selected and included the studies by browsing the full texts according to the eligibility criteria: 1) controlled studies assessing the ICA administration of ONFH animal models established by various methods; 2) ICA as monotherapy with medicament type, unrestricted dosage, route of administration, and time for the medicine application was involved in the treatment group, while normal saline or blank control was involved in the control group; and 3) the present study received bone pathology and/or bone mineral density [femur bone mineral density (F-BMD)] and/or bone histomorphometric parameters under micro-CT [(trabecular number (Tb.N), trabecular thickness (Tb.Th), trabecular separation (Tb.Sp), bone surface/total volume (BS/TV), bone volume/total volume (BV/TV), and bone volume (BV)] and index of bone metabolism and/or index of adverse reactions as the primary outcome measures, while the mechanisms of ICA for ONFH were selected as the second outcome measures. The study was excluded if it met the following criteria: 1) other types of studies (case reports, clinical trials, in vitro studies, reviews, etc.); 2) treatment with ICA in conjunction with other compounds; 3) compared with traditional Chinese medicine; 4) no primary outcome indicators involved or incomplete data; 5) no control group; 6) duplicate publications; and 7) not ONFH model.

### 2.3 Data extraction

A predefined form was separately executed by Jie Xu and Wei Deng to extract the information from the included studies. Details included were author and publication dates, animal data, modeling methods, the use of anesthesia, the therapeutic regimen of treatment and control group, and primary and/or secondary outcomes. Only data from the ONFH group and ICA + ONFH groups were included for analysis. When the measurement results of gradient doses for different drug treatments or at different times are displayed, only the highest dose and final measurement data are included for analysis.

### 2.4 Risk of bias in individual studies

The CAMARADES 10-item quality checklist (Macleod et al., 2004) with minor modification (D: blinded induction of model (group randomly after modeling); F: use of anesthetic without significant protective and toxic effects on bones; G: appropriate animal model (aged, hyperlipidemia, hypertensive, or diabetes); and I: compliance with animal welfare regulations [including three or more of the following points: preoperative anesthesia, postoperative analgesia, nutrition, disinfection, environment temperature, environment humidity, circadian rhythm, and euthanasia]) was used to assess the study quality separately by Xun Zhu and Deyu Han.

### 2.5 Statistical analysis

We utilized RevMan 5.3 software for data analysis where possible; otherwise, a comparison among groups was performed. Standardized mean differences (SMDs) and 95% confidence intervals (95% CIs) were calculated to estimate the combined overall effect sizes when the outcomes were determined in various ways or the unit of measurement is different. Heterogeneity was assessed using the Cochran’s Q-statistic test and the I^2^-statistic test. Random (I^2^> 50%) or fixed-effects model (I^2^< 50%) was selected. To ensure the reliability of results, a sensitivity analysis was carried out, and potential publication bias was assessed by the visual funnel diagram and asymmetry test. A probability value P < 0.05 was considered significant. Moreover, in order to explore the impact of potential confounding factors on the estimates of the combined effect size, subgroup analyses were conducted in this study.

## 3 Results

### 3.1 Study selection

**FIGURE 1:**
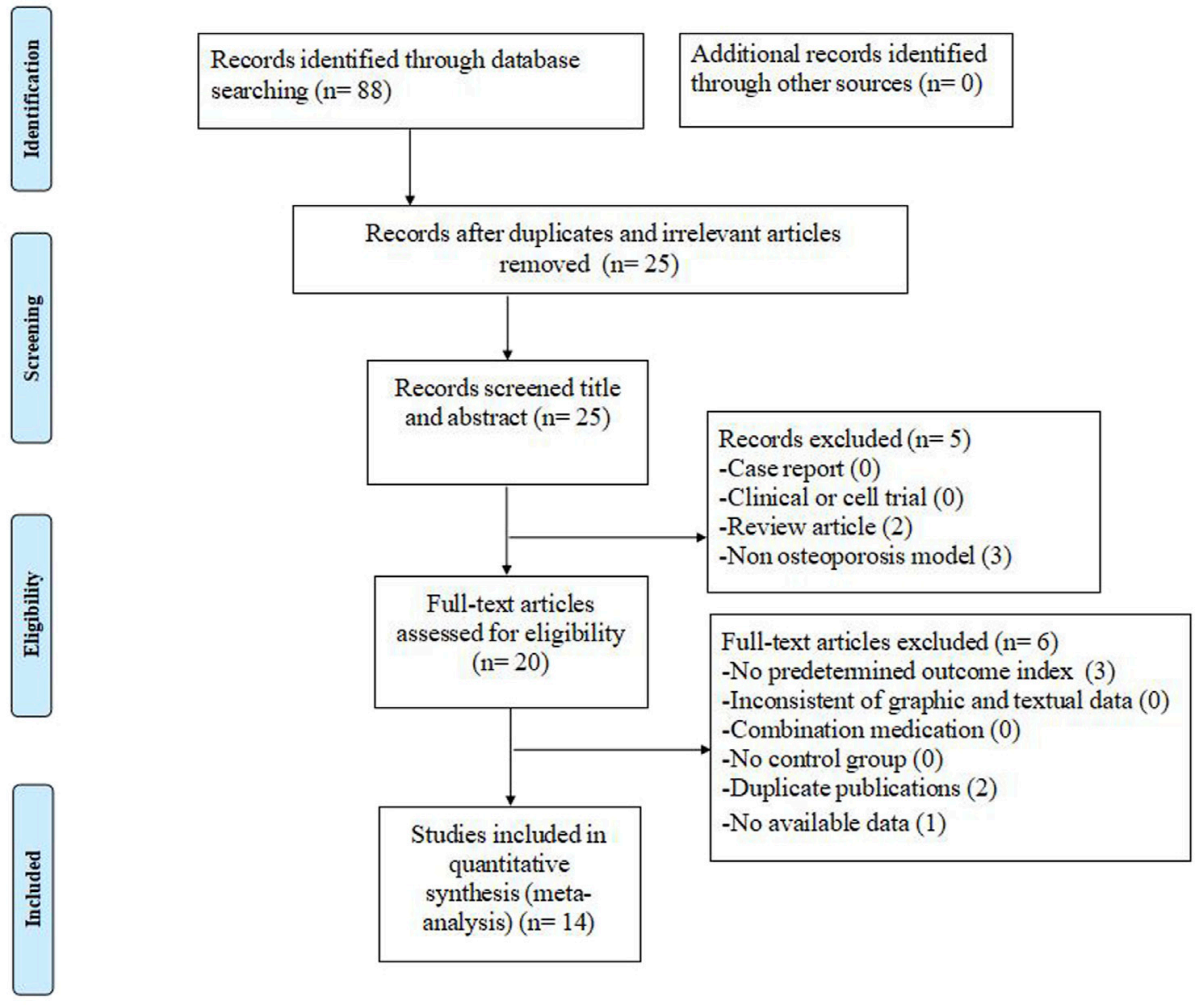
Summary of the process for identifying candidate studies.

The electronic search collected 93 studies ultimately. Sixty three duplicate or irrelevant studies were removed after screening titles and abstracts. After carefully reviewing the entire text, 11 studies were excluded because they were 1) clinical trials, 2) case reports, or 3) review articles. Among the remaining 19 full-text articles, 5 articles were excluded for at least one of the following reasons: 1) absence of a predetermined outcome index, 2) inconsistent graphic and textual dates, 3) combination medication, 4) no control group, 5) no available data, or 6) non-osteonecrosis of the femoral head model. Finally, 14 eligible studies (Yang et al., 2023; Shi et al., 2022; He et al., 2022; Jia et al., 2022; Wang et al., 2022; Yue et al., 2022; Liu, 2021; Shi et al., 2020; Peng et al., 2019; Yu et al., 2019; Xue, 2018; Huang et al., 2018; Meng et al., 2015; Xie et al., 2015) were included ultimately. The summary screening process is shown in Figure 1.

### 3.2 Characteristics of included studies

Three English studies and nine Chinese studies between 2015 and 2023 were identified. Sprague–Dawley (SD) rats (80.0%), Japanese white rabbits (9.4%), and New Zealand rabbits (10.6%) were used in total. Among which, 136 SD rats and 34 rabbits were treated with ICA, while 135 rats and 34 rabbits were treated as controls. Regarding the primary outcome measure, 10 studies (He et al., 2022; Jia et al., 2022; Yue et al., 2022; Liu, 2021; Shi et al., 2020; Peng et al., 2019; Yu et al., 2019; Xue, 2018; Huang et al., 2018; Xie et al., 2015) reported bone pathology; 6 studies (Shi et al., 2022; He et al., 2022; Wang et al., 2022; Liu, 2021; Huang et al., 2018; Meng et al., 2015) reported F-BMD; 9 studies (Yang et al., 2023; Shi et al., 2022; He et al., 2022; Jia et al., 2022; Wang et al., 2022; Liu, 2021; Shi et al., 2020; Xue, 2018; Huang et al., 2018) reported bone-related parameters under Micro-CT including Tb.N (Shi et al., 2022; He et al., 2022; Jia et al., 2022; Wang et al., 2022; Liu, 2021; Shi et al., 2020; Xue, 2018; Huang et al., 2018), Tb.Th (Shi et al., 2022; He et al., 2022; Jia et al., 2022; Wang et al., 2022; Liu, 2021; Shi et al., 2020; Xue, 2018; Huang et al., 2018), Tb.Sp (Shi et al., 2022; He et al., 2022; Jia et al., 2022; Wang et al., 2022; Liu, 2021; Shi et al., 2020; Xue, 2018), and BV/TV (Shi et al., 2022; He et al., 2022; Jia et al., 2022; Liu, 2021; Shi et al., 2020; Xue, 2018; Huang et al., 2018); 2 studies (Jia et al., 2022; Meng et al., 2015) reported the serum level of calcium and phosphorus; 1 study (Huang et al., 2018) reported the serum level of ALP; and 1 study (Yang et al., 2023) reported animal mortality. Relevant mechanism indicators such as vascular endothelial growth factor (VEGF), platelet endothelial cell adhesion (CD-31), nitric oxide (NO), osteoprotegerin (OPG), receptor activator of nuclear factor-κB (RANK), receptor activator of nuclear factor-κB ligand (RANKL), phosphatidylinositol 3-kinase (PI3K), threonine protein kinase (Akt), phosphorylated extracellular signal-regulated kinase-1 (p-ERK1), mitogen-activated protein kinases (p-p38), phosphorylated amino terminal kinase (p-JNK), Bax, B-cell lymphoma 2 (Bcl-2) and other detailed characteristics of the eligible studies are shown in Table 1.

**TABLE 1:** Characteristics of the included studies.

Study (years)	Species (sex, n = experimental/control group, weight, and age)	Model (method)	Anesthetic	Treatment group (method to astragal sides)	Control group	Outcome index (time)
Yang et al. (2023)	Half male and half female New Zealand rabbits (20/20, 2.5–3.0 kg, adult)	Injected with LPS (5 μg/kg) and MPS (20 mg/kg) sequentially	Ethyl carbamate	Gavage with ICA (calculated dose via experimental animal dose conversion) for 6 weeks	Gavage with an equal volume of NS for 6 weeks	1. Bone-related parameters under Micro-CT 2. Animal mortality
Shi et al. (2022)	Male SD rats (10/10, 230–270 g, 8 weeks old)	Intravenous injection of LPS (10 μg/kg) once every other day for two times and then intramuscular injection of MPS (40 mg/kg) once every other day for 10 times	Isoflurane	Gavage with ICA at 40 mg/kg for 6 weeks	Gavage with an equal volume of NS for 6 weeks	1. Bone-related parameters under Micro-CT (Tb.N, Tb.Th, Tb.Sp, and BV/TV) 2. BMD (femur) 3. Serum level of NO and VEGF 4. Bone level of PI3K, Akt, and p-Akt
He et al. (2022)	Male SD rats (20/20, 250–300 g, 12 weeks old)	Intraperitoneal injection of LPS (0.2 mg/kg) and then intramuscular injection of MPS (40 mg/kg) every day for 3 times	NM	Gavage with ICA at 100 mg/kg for 6 weeks	Gavage with an equal volume of NS for 6 weeks	1. Bone pathology 2. Bone-related parameters under Micro-CT (Tb.N, Tb.Th, and BV/TV) 3. BMD (femur) 4. Bone levels of OCN, TAZ, CD31, and VEGF 5. Femoral head vessel volume
Jia et al. (2022)	Male SD rats (12/12, 220–240 g, 2 months old)	Gavage with 46% of alcohol (10 mL/kg/d) for 24 weeks	Chloral hydrate	Gavage with ICA at 60 mg/kg for 8 weeks	Gavage with an equal volume of NS for 8 weeks	1. Bone pathology 2. Bone-related parameters under Micro-CT (Tb.N, Tb.Th, Tb.Sp, and BV/TV) 3. Serum and bone level of BMP-2, TGF-β, and bFGF 4. Bone levels of OPG, RANK, and RANKL 5. Number of activities, the total distance of activities, and maximum grasping of forcing 6. Serum levels of phosphorus and calcium
Wang et al. (2022)	Male SD rats (8/8, 270–310 g, 8 weeks old)	Intravenous injection of LPS with (2 mg/kg) for 2 times and then intramuscular injection of MPS (40 mg/kg) every day for 10 times	Sodium pentobarbital solution	Gavage with ICA (NM) for 8 weeks	Gavage with an equal volume of NS	1. Bone-related parameters under Micro-CT (Tb.N, Tb.Th, and Tb.Sp) 2. BMD (femur) 3. Bone levels of p-ERK1, p-p38, and p-JNK
Yue et al. (2022)	Female SD rats (30/30, NM, 8 weeks old)	Intravenous injection of LPS with (0.2ug/kg) for 2 times and then intramuscular injection of MPS (40 mg/kg) every day for 3 times	NM	Gavage with ICA with 60 mg/kg for 4 weeks	Gavage with an equal volume of NS for 4 weeks	1. Bone pathology 2. Width of trabecular, the percentage of the area of trabeculae, and the ratio of empty lacunae 3. Bone level of mi-RNA-23b
Liu (2021)	Female SD rats (10/10, NM, 8 week-old)	1. Intravenous injection of LPS with (0.2ug/kg) for 2 times and then intramuscular injection of MPS (40 mg/kg) every day for 3 times 2. Each rat was injected into the proximal femoral medullary cavity with 10ul purified adenovirus overexpression or interference vector (virus titer 1 × 10^11^ PFU/mL) on days 0 and 14	Sodium pentobarbital solution	Gavage with ICA with 60 mg/kg for 4 weeks	Gavage with an equal volume of NS for 4 weeks	1. Bone pathology 2. Bone-related parameters under Micro-CT (Tb.N, Tb.Th, Tb.Sp, BV, TV, and BV/TV) 3. BMD (femur) 4. Bone levels of CD31, miR-23b, SEMA6A, Sprouty2, and VEGF 5. Bone level of the ratio of CD4+/CD8+
Shi et al. (2020)	Female and male SD rats (18/16, 2.5–3.0 kg, NM)	Intravenous injection of LPS with (5 μg/kg) once and then intramuscular injection of MPS (20 mg/kg) every day for 3 times	Urethane	Gavage with ICA with 10 mL for 6 weeks	Gavage with an equal volume of NS for 6 weeks	1. Bone pathology 2. Incidence of osteonecrosis 3. Bone-related parameters under Micro-CT (Tb.N, Tb.Th, Tb.Sp, and BV/TV)
Peng et al. (2019)	Male New Zealand rabbits (9/9, 2.6–3.0 kg, 3.0–3.5 months old)	Intravenous injection of horse serum with (20 mL/kg) every week for 3 times and then intramuscular injection of DXM (10 mg/kg) every day for 4 times	NM	Implanted with three-dimensional printing β-TCP scaffold loaded with ICA	Implanted with autologous bone	1. Bone pathology 2. Number of osteoclasts and osteoblasts 3. Bone level of VEGF
Yu et al. (2019)	Male SD rats (10/10, 260 ± 20 g, NM)	By intraperitoneal injection of LPS (10 μg/kg) every day for two times and then intramuscular injection of MPS (40 mg/kg) three times a day for 3 days	Sodium pentobarbital solution	Gavage with ICA with 60 mg/kg for 6 weeks	Gavage with an equal volume of NS for 6 weeks	1. Bone pathology 2. Bone level of CD31 3. Angiography: total vessel volume 4. Bax and Bcl-2 5. VEGF
Xue (2018)	Male New Zealand rabbits (9/9, 2.6–3.0 kg, 3.0–3.5 months old)	Intravenous injection of horse serum with (20 mL/kg) every week for 3 times and then intramuscular injection of DXM (10 mg/kg) every day for 4 times	Chloral hydrate	Implanted with three-dimensional printing β-TCP scaffold loaded with ICA	Implanted with autologous bone	1. Bone pathology 2. Bone-related parameters under Micro-CT (Tb.N, Tb.Th, Tb.Sp, and BV/TV) 3. Bone level of VEGF
Huang et al. (2018)	Female SD rats (10/10, NM, 3 weeks old)	Intravenous injection of LPS (10 μg/kg) every day for two times and then intramuscular injection of MPS (20 mg/kg) every day for 3 times	NM	Intravenous injection of ICA with 30 mg/kg for 12 weeks	Intravenous injection of an equal volume of NS for 12 weeks	1. Bone pathology 2. Bone-related parameters under Micro-CT (Tb.N, Tb.Th, BS/TV, and BV/TV) 3. BMD (femur) 4. Bone levels of Runx2 and PPARγ 5. Serum levels of ALP and TG
Meng et al. (2015)	Male and female SD rats (8/9, 2.5–3.0 kg, NM)	Intravenous injection of horse serum for two times (10 mL/kg once, 6 mL/kg once) and then intraperitoneal injection of MPS (45 mg/kg) every day for 3 times	Sodium pentobarbital solution	Gavage with ICA with 10 mL for 8 weeks	Gavage with an equal volume of NS for 8 weeks	1. BMD (femur) 2. Serum levels of calcium and phosphorus
Xie et al. (2015)	Male Japanese white rabbit (16/16, 2.8–3.4 kg, NM)	Intramuscular injection of MPS (20 mg/kg) for 2 weeks	NM	Implanted with three-dimensional printing β-TCP scaffold loaded with ICA	Implanted with autologous bone	1. Bone pathology 2. Histological grading score 3. Radiological grading score 2. Bone level of VEGF 3. X-ray and CT scan of femur

LPS, lipopolysaccharide; DXM, dexamethasone; ICA, icariin; β-TCP, β-tricalcium; Tb.N, trabecular number; Tb.Th, trabecular thickness; Tb.Sp, trabecular separation; BV/TV, bone volume/total volume; BS/TV, bone surface/total volume; WBC, white blood cell; RBC, red blood cell; ALT, glutamic–pyruvic transaminase; AST, glutamic oxaloacetic transaminase; Ccr, creatinine clearance rate; BMP-2, bone morphogenetic protein-2; VEGF, vascular endothelial growth factor; MPS, methylprednisolone; NS: normal saline; BMD, bone mineral density; OCN, osteocalcin; TAZ, transcriptional co-activator with PDZ-binding motif; CD31, platelet endothelial cell adhesion; SEMA, semaphorin; TGF-β, transcriptional growth factor; bFGF, basic fibroblast growth factor; OPG, osteoprotegerin; RANKL, receptor activator of nuclear factor-κB, ligand; RANK, receptor activator of nuclear factor-κB; p-ERK1, phosphorylated extracellular signal-regulated kinase-1; p-p38, mitogen-activated protein kinases; p-JNK, phosphorylated amino terminal kinase; Runx2, runt-related transcriptional factor 2; PPARγ, peroxisome proliferator-activated receptor γ; ALP, alkaline phosphatase; TG, triglyceride; TCP, tricalcium phosphate; Bcl-2: B-cell lymphoma 2; PI3K, phosphatidylinositol 3-kinase; Akt, threonine protein kinase; NO, nitric oxide.

### 3.3 Study quality

The quality score for the included articles ranges from 1/10 to 6/10, with an average of 4.5. Table 2 shows the author’s assessment of each bias risk item for each included study.

**TABLE 2:** Risk of bias of the included studies.

Study	A	B	C	D	E	F	G	H	I	J	Total
Yang et al. (2023)	√	√	√			√			√	√	6
Shi et al. (2022)	√	√	√			√			√	√	6
He et al. (2022)	√	√	√								3
Jia et al. (2022)	√	√	√	√		√				√	6
Wang et al. (2022)	√	√	√			√					4
Yue et al. (2022)	√	√	√		√				√		5
Liu (2021)		√	√			√	?		√		4
Shi et al. (2020)	√	√	√			√			√	√	6
Peng et al. (2019)	√		√	√	√					√	5
Yu et al. (2019)	√		√			√				√	4
Xue (2018)			√	√	√	√			√		5
Huang et al. (2018)	√		√							√	3
Meng et al. (2015)	√										1
Xie et al. (2015)	√		√	√	√				√		5

Note: Studies fulfilling the criteria of: A: peer reviewed publication; B: control of temperature; C: random allocation to treatment or control; D: blinded induction of model (group randomly after modeling); E: blinded assessment of outcome; F: use of anesthetic without significant protective and toxic effects on bones; G: appropriate animal model (aged, hyperlipemia, hypertensive, or diabetes); H: sample size calculation; I: compliance with animal welfare regulations (including three or more of the following points: preoperative anesthesia, postoperative analgesia, nutrition, disinfection, environment temperature, environment humidity, circadian rhythm, and euthanasia); J: statement of potential conflict of interests.

### 3.4 Effectiveness

#### 3.4.1 Bone pathology

**FIGURE 2:**
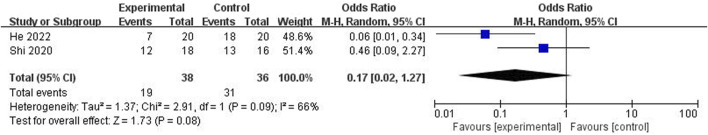
Forest plot: effects of icariin for decreasing the incidence of osteonecrosis compared with the control group.

Bone pathology was reported in 10 studies (He et al., 2022; Jia et al., 2022; Yue et al., 2022; Liu, 2021; Shi et al., 2020; Peng et al., 2019; Yu et al., 2019; Xue, 2018; Huang et al., 2018; Xie et al., 2015), 6 of which (He et al., 2022; Jia et al., 2022; Yue et al., 2022; Liu, 2021; Shi et al., 2020; Yu et al., 2019) found that ICA could reduce bone lacuna emptying, neatly arrange the bone trabeculae, narrow the bone trabecular space, and reduce osteoblast apoptosis. Four studies (Peng et al., 2019; Xue, 2018; Huang et al., 2018; Xie et al., 2015) reported that ICA could mature osteoblasts of ONFH and decrease chondroid bone matrix and fibrous connective tissue in bones. Meta-analysis of two studies (He et al., 2022; Shi et al., 2020) showed the significant effect of ICA for the decreasing incidence of osteonecrosis (n = 74; OR 0.17; 95% CI (0.06, 0.52); P < 0.00001; heterogeneity: Tau^2^ = 1.37; χ^2^ = 2.91; I^2^ = 66%; Figure 2) compared with the control group.

#### 3.4.2 Bone-related parameters under imageology

**FIGURE 3:**
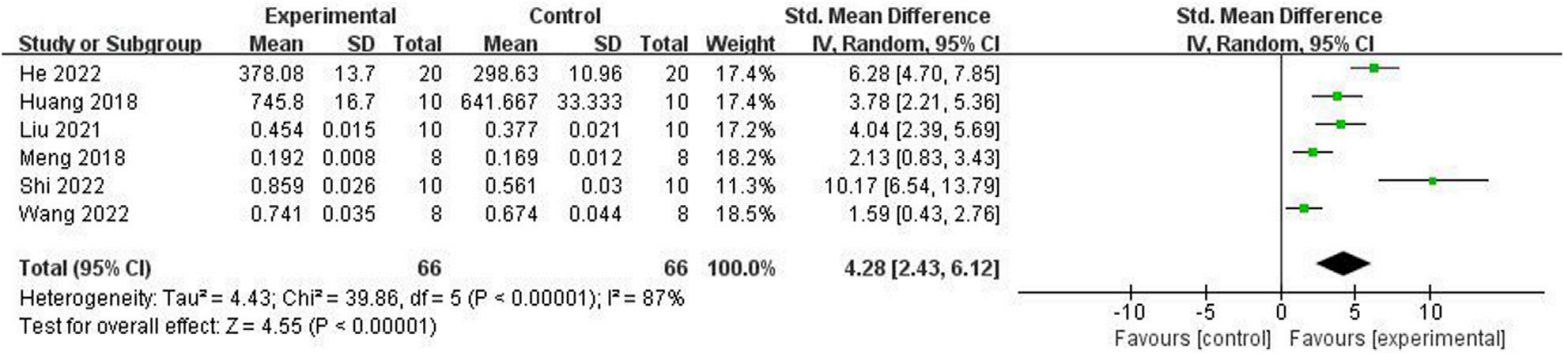
Forest plot: effects of icariin for increasing the femur bone mineral density (F-BMD) compared with the control group.

Relying on dual-energy X-ray absorptiometry, meta-analysis of six studies (He et al., 2022; Wang et al., 2022; Liu, 2021; Shi et al., 2020; Huang et al., 2018; Meng et al., 2015) showed the significant effect of ICA for increasing F-BMD (n = 132; SMD 4.28; 95% CI (2.43, 6.12); P < 0.00001; heterogeneity: Tau^2^ = 4.43; χ^2^ = 39.86; I^2^ = 87%; Figure 3) compared with the control group.

**FIGURE 4:**
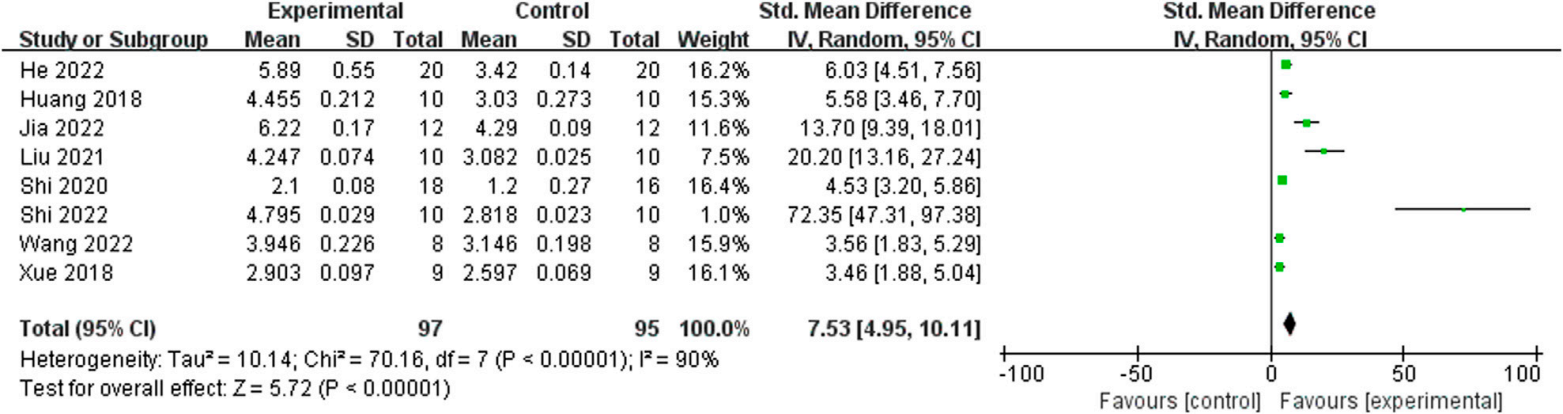
Forest plot: effects of icariin for increasing trabeculae linear density (Tb.N) compared with the control group.

**FIGURE 5:**
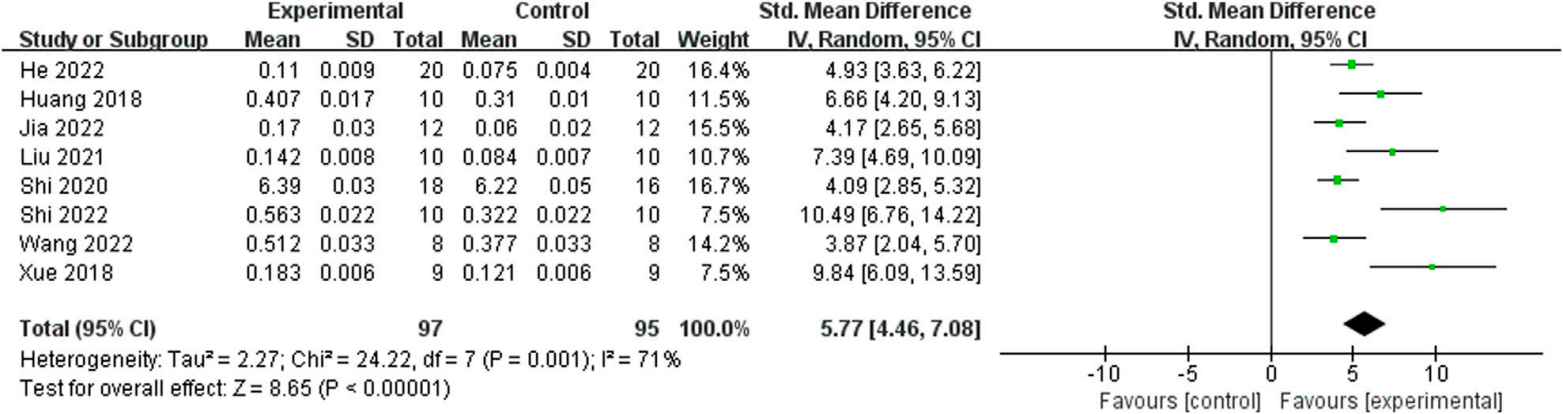
Forest plot: effects of icariin for increasing trabeculae thickness (Tb.Th) compared with the control group.

**FIGURE 6:**
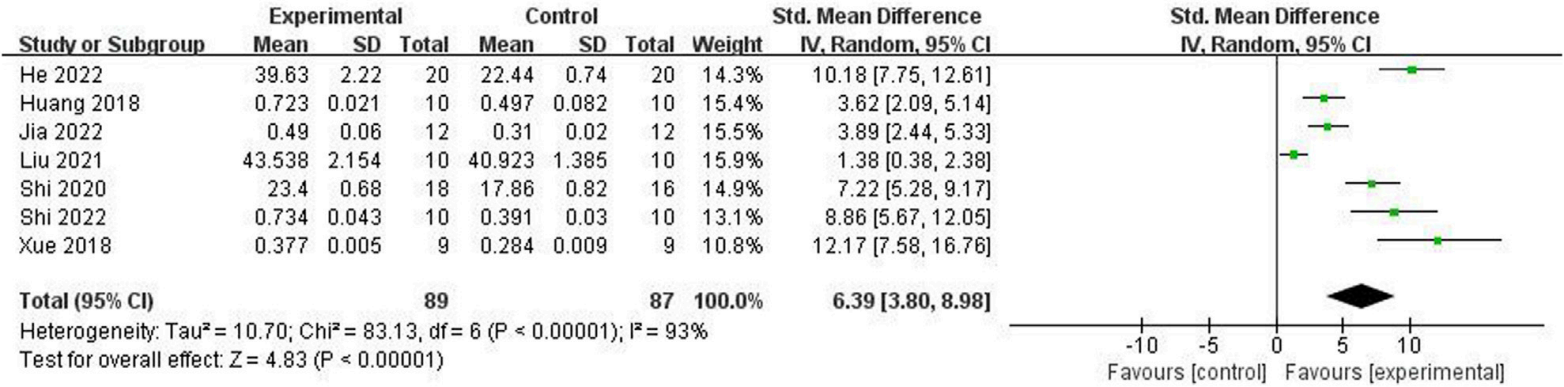
Forest plot: effects of icariin for increasing the bone volume/total volume (BV/TV) compared with the control group.

**FIGURE 7:**
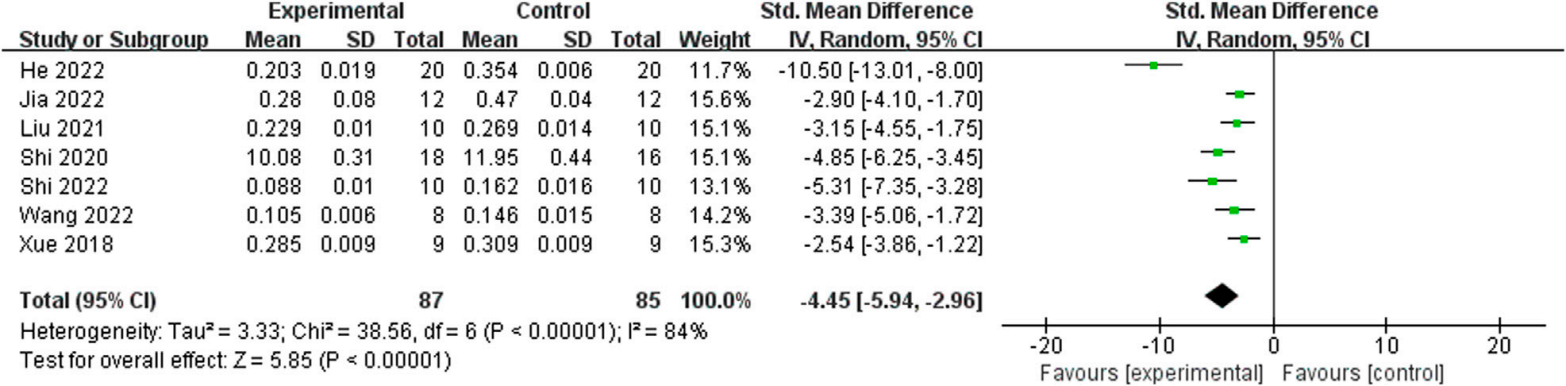
Forest plot: effects of icariin for decreasing trabecular separation (Tb.Sp) compared with the control group.

**FIGURE 8:**
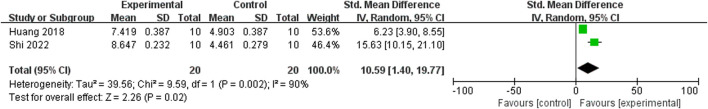
Forest plot: effects of icariin for increasing the bone surface/total volume (BS/TV) compared with the control group.

Relying on Micro-CT, meta-analysis of eight studies (Shi et al., 2022; He et al., 2022; Jia et al., 2022; Wang et al., 2022; Liu, 2021; Shi et al., 2020; Xue, 2018; Huang et al., 2018) showed the significant effect of ICA increasing Tb.N (n = 192; SMD 7.53; 95% CI (4.95, 10.11); P < 0.00001; heterogeneity: Tau^2^ = 10.14; χ^2^ = 70.16; I^2^ = 90%; Figure 4) and Tb.Th (n = 192; SMD 5.77; 95% CI (4.46, 7.08); Tau^2^ = 2.27; χ^2^ = 24.22; I^2^ = 71%; Figure 5). Seven studies (Shi et al., 2022; He et al., 2022; Jia et al., 2022; Wang et al., 2022; Liu, 2021; Shi et al., 2020; Xue, 2018) showed the significant effect of ICA increasing BV/TV (n = 176; SMD 6.39; 95% CI (3.80, 8.98); P < 0.00001; heterogeneity: Tau^2^ = 10.70; χ^2^ = 83.13; I^2^ = 93%; Figure 6) and decreasing Tb.Sp (n = 87; SMD -4.45, 95% CI (−5.94, −2.96); P < 0.00001; heterogeneity: Tau^2^ = 3.33; χ^2^ = 38.56; I^2^ = 84%, Figure 7). Two studies (Huang et al., 2018; Shi et al., 2022) reported that ICA could increase BS/TV (n = 40; SMD 10.59; 95% CI (1.47, 19.77); P = 0.02; χ^2^ = 9.59; I^2^ = 90%; Figure 8). Liu et al. reported that ICA could improve BV with no statistically significant differences (Liu, 2021).

#### 3.4.3 Animal mortality

One study (Yang et al., 2023) reported that four animals in the GC group and two animals in the ICA group eventually died, suggesting that ICA may not increase the mortality rate.

#### 3.4.4 Mechanism indicators

**FIGURE 9:**
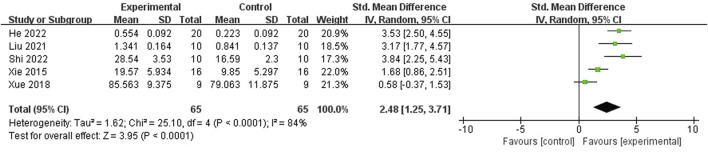
Forest plot: effects of icariin for improving the bone level of vascular endothelial growth factor (VEGF) compared with the control group.

**FIGURE 10:**
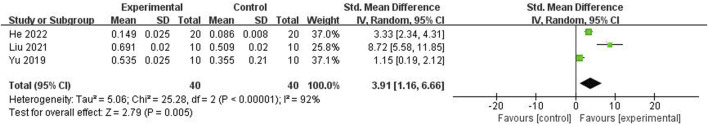
Forest plot: effects of icariin for improving the bone level of platelet endothelial cell adhesion (CD-31) compared with the control group.

ICA was reported to improve the bone levels of VEGF (He et al., 2022; Liu, 2021; Shi et al., 2022; Xue, 2018; Xie et al., 2015) (n = 130; SMD 2.48; 95% CI (1.25, 3.71); P < 0.0001; Tau^2^ = 1.62; χ^2^ = 25.10; I^2^ = 84%; Figure 9), CD31 [20, 24, 27] (n = 80; SMD 3.91; 95% CI (1.16, 6.66); P < 0.00001; Tau^2^ = 5.06; χ^2^ = 25.28; I^2^ = 92%; Figure 10), and NO (Shi et al., 2022), and BMP-2, OPG, RANK, RANKL (Jia et al., 2022), TAZ, Runx2, and CTGF (He et al., 2022) compared with the control group. In addition, Yu et al. (2019) reported that ICA increased the protein level of Bax and decreased the protein level of Bcl-2.

#### 3.4.5 Sensitivity analyses and subgroup analysis

**FIGURE 11:**
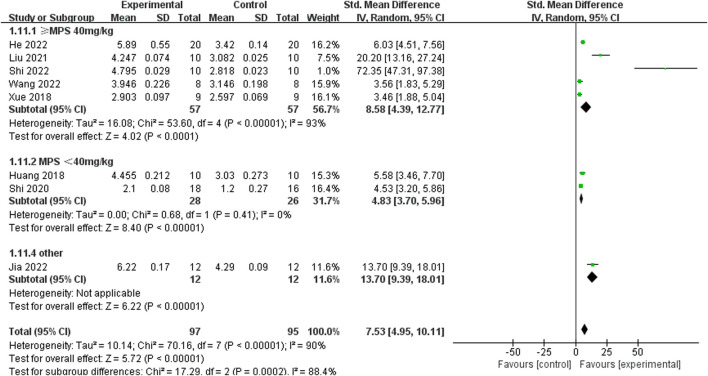
Subgroup analysis: the effect size of the different molding methods on the trabecular number in ONFH animals treated with ICA.

Considering the remarkable heterogeneity of the results, sensitivity analyses and subgroup analysis of primary outcome measures (F-BMD, Tb.N, Tb.Th, BV/TV, and Tb.Sp) were conducted. Sensitivity analyses showed that the heterogeneity and standard mean difference (SMD) of any of the above indicators did not decline significantly after eliminating any study. By perusing the characteristics of each including study, the potential confounding factors (different modeling methods, varying doses of ICA, and different administration methods) might increase the heterogeneity through the hierarchical analysis of Tb.N. In the subgroup analysis of different molding methods, the model effect induced by high-dose MPS (≥40 mg/kg) injection exhibited better results compared to the low-dose MPS (<40 mg/kg) injection (SMD 8.58 vs. SMD 4.83, P < 0.0001, and Figure 11), and SMD 13.7 in other modeling methods group (ONFH caused by alcohol) (Jia et al., 2022) was obviously different from that of the hormone model group. In addition, the heterogeneity of the low-dose MPS group decreased significantly, suggesting that different modeling methods might be sources of high heterogeneity. In the subgroup analysis of varying doses of ICA and different administration methods, although there are slight differences in effect values among different groups, the heterogeneity between different groups is still high, suggesting that these two factors are not the main source of heterogeneity.

## 4 Discussion

### 4.1 Summary of evidence

The first-ever preclinical comprehensive evidence includes a batch of studies with acceptable quality to estimate the efficacy and multiple mechanisms of ICA in animal models of ONFH.

### 4.2 Limitations

**FIGURE 12:**
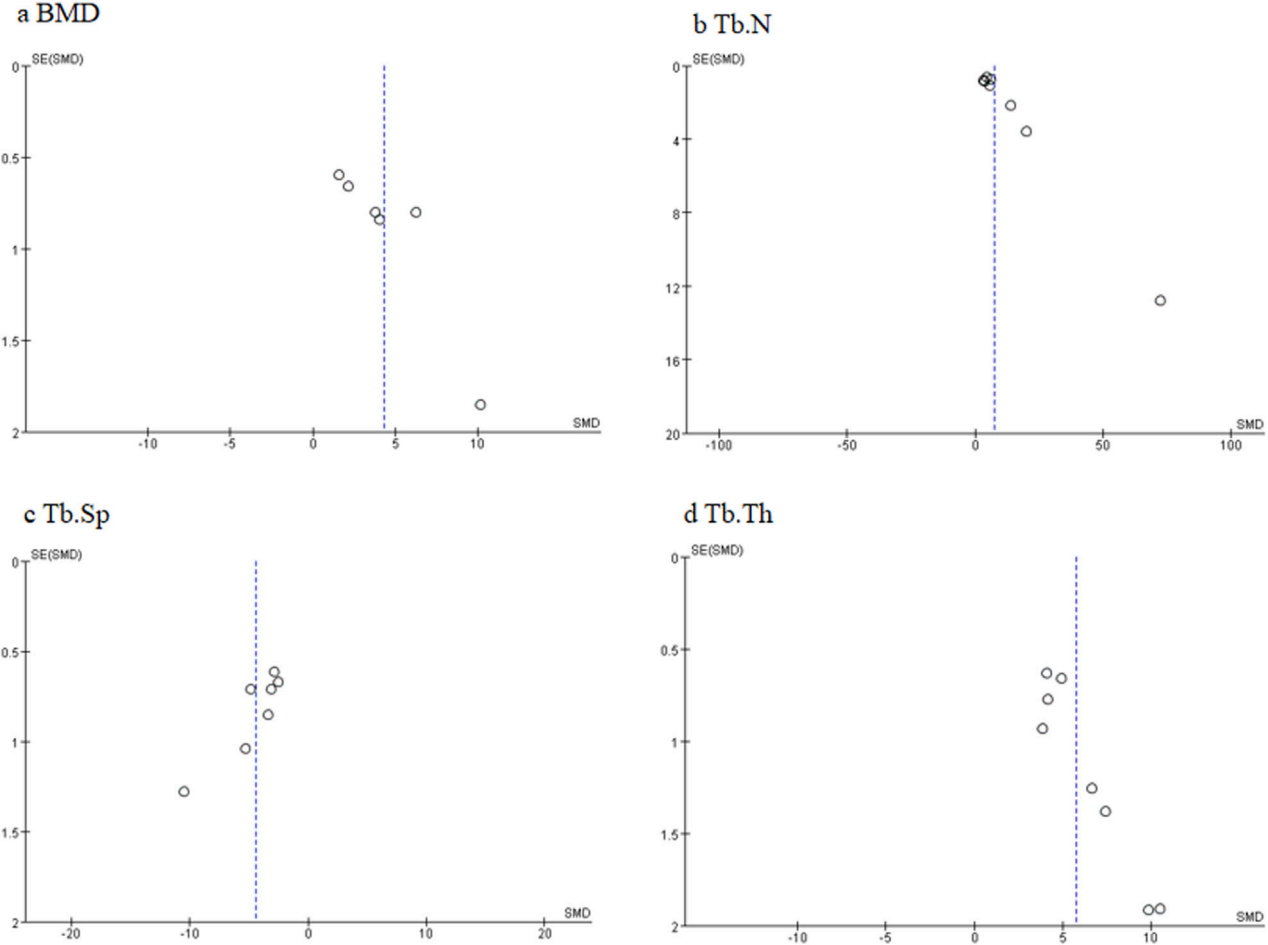
Funnel plots of different primary outcome measures: **(A)** bone mineral density (BMD); **(B)** trabecular number (Tb.N); **(C)** trabecular separation (Tb.Sp); and **(D)** trabecular thickness (Tb.Th).

Some limitations are listed as follows: 1) the funnel plots shown in Figure 12 demonstrated the potential publication bias in this research field, suggesting studies with negative or null effects that are missing. Publication bias is caused by multiple factors such as researchers and journal editors preferring positive results rather than negative or inconclusive results (Wolfgang., 2007). Thus, the effect of ICA on ONFH cannot be excluded from the overall overestimation of effect sizes and efficacy, which may weaken the validity of conclusions. We call for some negative studies and protocols on ICA for ONFH to be submitted and accepted those after rigorous review. 2) The absence of sample size calculation may impact the credibility of findings. 3) ONFH generally occurs in patients with medical issues such as old age, diabetes, hypertension, and hyperlipidemia, but none of the included studies used an ONFH model with relevant comorbidities. To facilitate the clinical translation of ICA for ONFH, future research should focus on evaluating whether ICA still plays a similar role in ONFH under mixed factors. 4) Among these studies, there is a general lack of safety, toxicity assessments, and reflections on the limitations to experiments.

### 4.3 Implication

Defects in the aspects of the core standards of study design such as randomization, blinding, and sample size calculation are fatal blows to the credibility of a basic research project (Moher et al., 2015). The blinding methods in animal model establishment and the outcome assessment were usually seen as technical difficulties for most studies. It is commendable that four studies (Jia et al., 2022; Peng et al., 2019; Xue, 2018; Xie et al., 2015) report the blinding protocols (group randomization after modeling) and four studies (Yue et al., 2022; Peng et al., 2019; Xue, 2018; Xie et al., 2015) report the blinded assessment of the outcome (random selection of animals for result evaluation). Although most articles mention random grouping in all studies, only five studies (Shi et al., 2022; Wang et al., 2022; Yue et al., 2022; Liu, 2021; Shi et al., 2020) provide information on reasonable random methods including the randomization sequence generated from a random number table, calculator, or computerized random-number generator. Future articles should avoid allocating participants according to the date of birth, their hospital record numbers, or the date on which they were invited to participate as these are not considered adequate. Drawing straws or coin-tossing in front of the participant to decide grouping were also considered ineligible randomization techniques (Wu et al., 2009). In addition, the sample size is one of the first and foremost questions to be answered when designing a study, and increasing the sample size calculation improves the credibility of the literature (Zhang and Hartmann, 2023). A sample size calculation could avoid the waste of resources caused by oversizing and the imprecision of the study result by undersizing. For specific steps, please refer to the study by Gupta et al. (2016). Future research studies also need to include more experimental animals with comorbidities such as obesity, venerable age, hypertension, hyperlipidemia, or other risk factors which are consistent with the physiology of patients with ONFH. In the field of ONFH research, the insufficient work on randomization and sample size calculation in the included studies needs to be taken seriously. The ARRIVE guidelines (Kilkenny et al., 2010) are suggested to be referred to in the future for designing experiments and reporting results.

High-quality methodologies of studies are the cornerstones of translating animal research into clinical drug treatments for human disease (García-Bonilla et al., 2012), among which the selection of animal modeling methods plays a pivotal role. Different modeling methods may be the source of high heterogeneity in the current research study. Currently, the three commonly used molding methods are as follows: 1) short term high-dose glucocorticoid administration is adapted in many of the including studies (He et al., 2022; Shi et al., 2020; Yue et al., 2022; Liu, 2021; Peng et al., 2019; Yu et al., 2019; Huang et al., 2018; Meng et al., 2015), whose main feature is that the experimental animals are disposed with a large dose of glucocorticoid in a short time. The usual operating step is to inject MPS intramuscularly at a dose of 20 mg/kg once a day for 2 weeks (Zhao et al., 2013; Kang et al., 2015). The method is fast in modeling and easy to operate, and the results of histopathology indicate that the success rate of this modeling method is 70%–75% in the previously included studies (Zhao et al., 2013; Kang et al., 2015). However, it cannot be ignored that the excessive dose of glucocorticoids may result in high animal mortality (Wang et al., 2021), which was reported to be 20% in the report by Kuribayashi et al. (2010). 2) Long-term, sustained low-dose glucocorticoid administration is designed to simulate the clinical long-term use of glucocorticoids, leading to ONFH. The usual operating step is to intravenously inject MPS at a dose of 7.5 mg/kg to animals once every 3 days for 6 weeks (Wang et al., 2021). The prior study found a modeling success rate of 85.7% and an animal mortality of 12.5% with good repeatability of experimental results (Wang et al., 2020), which is better than short-term high-dose glucocorticoid administration. Its biggest drawback is that it is not suitable for building a large number of models in a short time. 3) Intermittent administration is modified from the two abovementioned methods. Zhao et al. (2013) administered rats 8 mg/kg of prednisolone once a day intramuscularly to animals for 3 weeks and continued the injection for 5 weeks, following a 3-week rest period. Compared to long-term, sustained low-dose glucocorticoid administration, intermittent administration can further reduce the mortality rate of animals, and the degree of ONFH in the group of intermittent administration methods is much greater than that of long-term, sustained low-dose glucocorticoid administration. It is unfortunate that this method is not adopted in the studies included. The relatively long experimental period and poor repeatability due to the difference in intermittent time may limit the application of this method. In order to better simulate the inflammatory environment of the body before clinical ONFH, the adjuvant or allogeneic serum combined with the hormone modeling method has been considered a more suitable method for ONFH modeling. Studies (Mikami et al., 2010; Jia et al., 2017) have shown that ONFH is not only related to hormones but also to abnormalities in the immune system before the use of hormones. Therefore, the addition of adjuvants such as LPS or heterologous animal serum can cause immune responses to form abnormalities, leading to microcirculation disorders of local bone tissue, which will form ONFH quickly and efficiently. Only two (Jia et al., 2022; Xie et al., 2015) of our included articles modeled without LPS or heterologous animal series, bringing the model closer to the pathophysiology of human ONFH. Unfortunately, there are no studies that use the injection of LPS or heterologous serum combined with intermittent administration to construct models, which should be investigated more in the future, considering its potential higher success rates and lower mortality rates.

**FIGURE 13:**
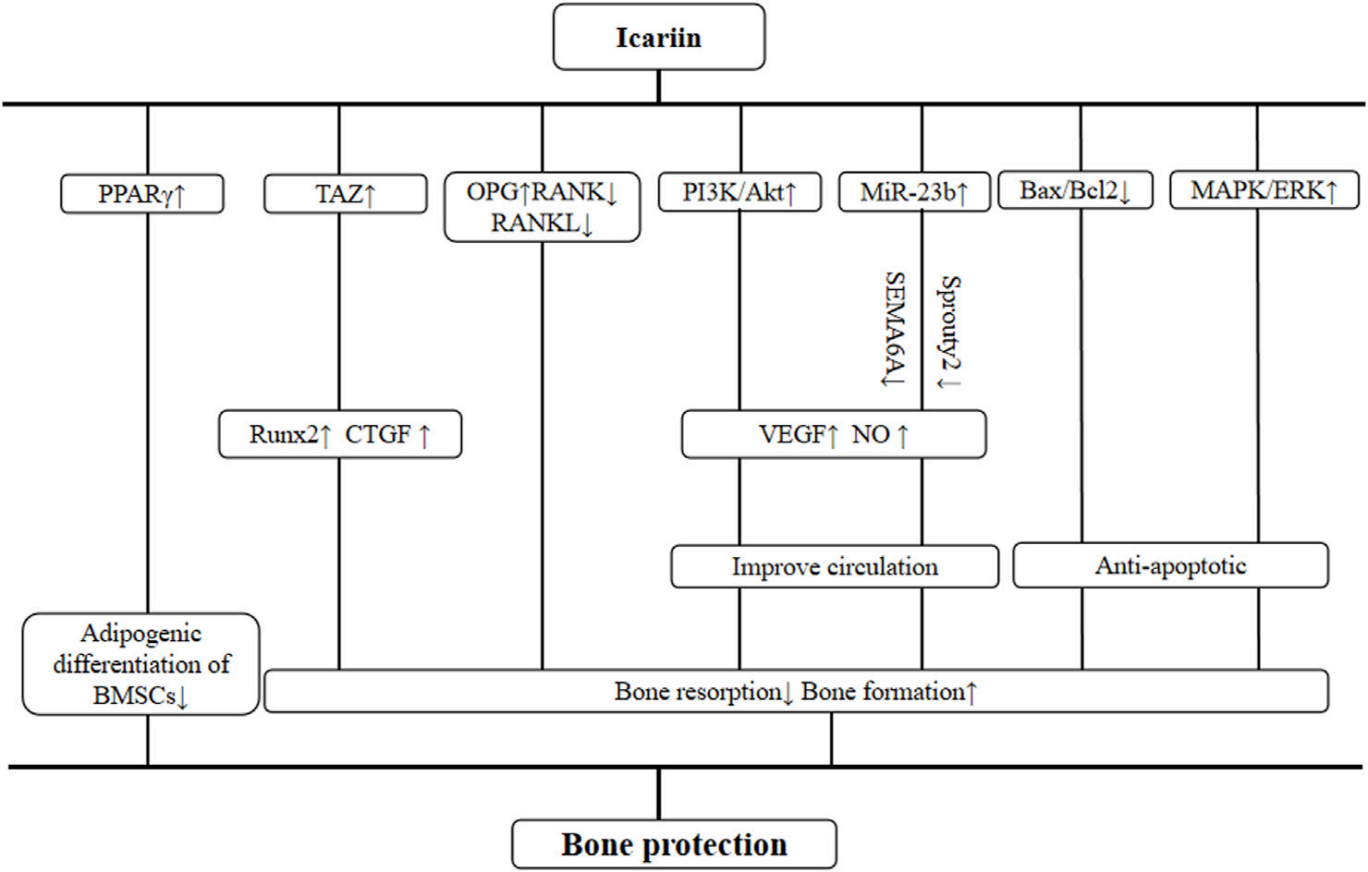
Schematic representation of osteoprotective mechanisms of icariin for osteonecrosis of the femoral head.

The systemic review of preclinical studies is conducive to the comprehensive understanding of pathological mechanisms of disease and pharmacological effects of drugs (Hackam and Redelmeier, 2006). We have summarized the possible mechanisms of ICA-mediated bone protection from current findings and listed them as follows: 1) three studies (Shi et al., 2022; Liu, 2021; Yu et al., 2019) have observed that the PI3k/Akt signaling pathway is activated by ICA. Shi et al. also reported the expression of VEGF, and NO was upregulated after the activation of the PI3K/Akt pathway, increasing vascular permeability to mediate vascular repair and regeneration. Both *in vivo* and *in vitro* experiments (Yue et al., 2022; Liu, 2021) have shown that ICA improved the inhibitory effect of hormones on miR-23b in bone microvascular endothelial cells (BMECs). MiR-23b, a non-coding miRNA that regulates angiogenesis, downregulated the expression of SEMA6A and Sprouty2 and upregulated the expression of VEGF (Liu, 2021; Yu et al., 2019; Xue, 2018). 2) Yu et al. (2019) reported that pre-treatment with ICA markedly reduced the expression of Bax and elevated the expression of Bcl-2 relative to the control group (P < 0.05). In addition, ICA affects bone cell apoptosis by promoting the MAPK/ERK signal pathway (Wang et al., 2022; Liu, 2021). 3) The OPG/RANK/RANKL pathway is an important signaling pathway found in recent studies (Fu et al., 2020; Sun et al., 2021) related to ONFH. ICA inhibits osteoclast activity by increasing OPG levels while reducing RANK and RANKL levels (Jia et al., 2022). In terms of osteogenesis, ICA may increase the expression of runt-related transcription factor 2 (Runx2) and connective tissue growth factor (CTGF) by promoting the transcriptional co-activator with PDZ binding motif (TAZ) concentration, thereby weakening the regulatory effect of dexamethasone on BMSC proliferation and osteogenic differentiation (He et al., 2022). 4) ICA can inhibit the adipogenic differentiation of BMSCs treated with MPS through the PPARγ-mediated pathway (He et al., 2022; Huang et al., 2018), which is the master regulator of adipogenesis and has been shown to have anti-osteoblastogenic effects (James, 2013). The mechanism diagram is summarized in Figure 13.

### 4.4 Conclusion

The present study provides evidence that ICA is capable of partially reversing ONFH in animal models probably via promoting angiogenesis, inhibiting apoptosis, and regulating osteogenic and osteoclast activities. Randomization, blinding, and sample size calculation should be the focus of ONFH research in the future. It reveals the possibility of developing ICA as a drug for the clinical treatment of ONFH.

## Data Availability

The raw data supporting the conclusions of this article will be made available by the authors, without undue reservation.

## Appendix 1. PubMed search strategy.

**Table:**

1. PubMed	
#1	Search (Icariin [Title/Abstract])
#2	Search (((Femoral head necrosis) OR (Femur head necrosis)) OR (Osteonecrosis)) OR (Osteonecrosis of the Femoral Head)
#3	#1 AND #2
#4	Filters: publication date to 2024/11/12. Items found: 19
