# Supplementary material for: Potential efficacy and mechanisms of icariin for the animal model of osteonecrosis of the femoral head
Source: Front Pharmacol. 2025 Feb 19;16:1508971. doi: 10.3389/fphar.2025.1508971 (PMC11880249; doi:10.3389/fphar.2025.1508971)
Supplement: Supplementary file 1 [file Table1.DOCX]

Pubmed search strategy

| 1. Pubmed | |
| --- | --- |
| #1 | Search (Icariin [Title/Abstract]) |
| #2 | Search (((Femoral head necrosis) OR (Femur head necrosis)) OR (Osteonecrosis)) OR (Osteonecrosis of the Femoral Head) |
| #3 | #1 AND #2 |
| #4 | Filters: Publication date to 2024/11/12.  Items found: 19 |
